# Obituary

**Published:** 1895-01

**Authors:** 


### Obituary.

Dr. Louis Alexander Bull, of Buffalo, died of diphtheria, at his residence, No. 665 West Ferry street, on Friday, November 30, 1894, aged thirty-seven years. He was taken ill six days before his death, and the community was shocked to learn of his demise almost before it had heard of his illness.

Dr. Bull was well known as a specialist in laryngology, having built up a successful practice in that branch of medicine. At the time of his death he was a member of the Buffalo Homeopathic Medical Society. At one time he was president of this body. He was on the consulting staff of the Wilcox Private Hospital, and was also on the staffs of the Erie County Hospital and the Homeopathic Hospital. Dr. Bull was a member of the Civil Service Commission, and was one of the originators as well as the secretary of the Twenty-fourth Ward Good Government Club. He was also the treasurer of the Buffalo Society of Natural Sciences. Dr. Bull was a prominent member of the Church of Our Father, being one of the trustees. He was also secretary of the literary society of the church.

He was the only son of Dr. Alexander T. Bull, of this city, who, together with the surviving widow and children and a sister, receives the sympathy of a wide circle of friends in this sadly sudden affliction.

---

Dr. F. L. Sim, editor of the *Memphis Medical Monthly*, died at his home, in Memphis, Tenn., on Tuesday, November 27, 1894, aged sixty years. He was also professor of the principles and practice of medicine in the Memphis Hospital Medical College, and was executive officer of the faculty. Dr. Sim was one of the leading physicians of the South and will be greatly missed by a large circle of friends. Mrs. Sim and a niece survive. His health had been failing for some months past, but the end came quickly and almost unexpectedly.

His colleagues in the faculty and various societies have adopted suitable memorials and the medical journals are paying tribute to his memory.

